# Laparoscopic Repair of Postoperative Perineal Hernia

**DOI:** 10.1155/2010/126483

**Published:** 2010-08-09

**Authors:** Stephen Ryan, Dara O. Kavanagh, Paul C. Neary

**Affiliations:** Department of Colorectal Surgery, The Adelaide and Meath Hospital incorporating the National Children's Hospital, 24 Dublin, Ireland

## Abstract

Perineal hernias are infrequent complications following abdominoperineal operations. Various approaches have been described for repair of perineal hernias including open transabdominal, transperineal or combined abdominoperineal repairs. The use of laparoscopic transabdominal repair of perineal hernias is not well-described. We present a case report demonstrating the benefits of laparoscopic repair of perineal hernia following previous laparoscopic abdominoperineal resection (APR) using a nonabsorbable mesh to repair the defect. We have demonstrated that the use of laparoscopy with repair of the pelvic floor defect using a non absorbable synthetic mesh offers an excellent alternative with many potential advantages over open transabdominal and transperineal repairs.

## 1. Introduction

Postoperative perineal hernia may be defined by the protrusion of intra-abdominal viscera through a defect in the pelvic floor into the perineal region. Perineal hernias are infrequent complications following abdominoperineal operations with a recent retrospective study estimating the prevalence to be 0.34% [1]. The most common presenting symptoms are that of pain and a dragging sensation or discomfort on standing, but urinary symptoms, intestinal obstruction, or perineal skin breakdown may also occur. Various approaches have been described for repair of perineal hernias including open transabdominal, transperineal, or combined abdominoperineal repairs [2–5]. 

The use of laparoscopic transabdominal repair of perineal hernias is not well-described in the medical literature to date [6–10]. Concerns about the insertion of intra-abdominal meshes to close peritoneal defects are largely founded upon the risk of adhesions, mesh infection, and the potential for fistula formation. These concerns, however, are tempered by the development of new synthetic meshes used in the increasingly popular technique of laparoscopic incisional hernia repairs. These meshes are reported to reduce the incidence of mesh-related morbidity and facilitate a minimally invasive approach to reconstructive surgery for large abdominal wall defects with the recognised benefits of laparoscopy. We present a case report demonstrating the benefits of laparoscopic repair of postoperative perineal hernia following laparoscopic abdominoperineal resection (APR) using a nonabsorbable mesh to repair the defect.

## 2. Case Presentation

A 69-year-old man presented with a history of rectal bleeding. Digital rectal examination revealed a palpable, fungating rectal lesion. Following EUA and biopsy, the lesion was confirmed as squamous cell carcinoma. Staging computed tomography scans did not reveal any distant metastasis. Following neoadjuvant chemoradiotherapy a preoperative MRI showed reduction in tumour size from 8.5 to 4.7 cm. The patient successfully underwent laparoscopic abdominoperineal resection of the rectum and formation of colostomy. He was discharged day 6 postoperatively with wounds intact.

Histology revealed an invasive poorly differentiated squamous cell carcinoma with extension into the perianal soft tissues. Lymphovascular space and perineural invasion were identified but all resection margins were negative for tumour. The tumour was classified as a yT_2_N_0_M_0_ lesion. 

**Figure 1:**
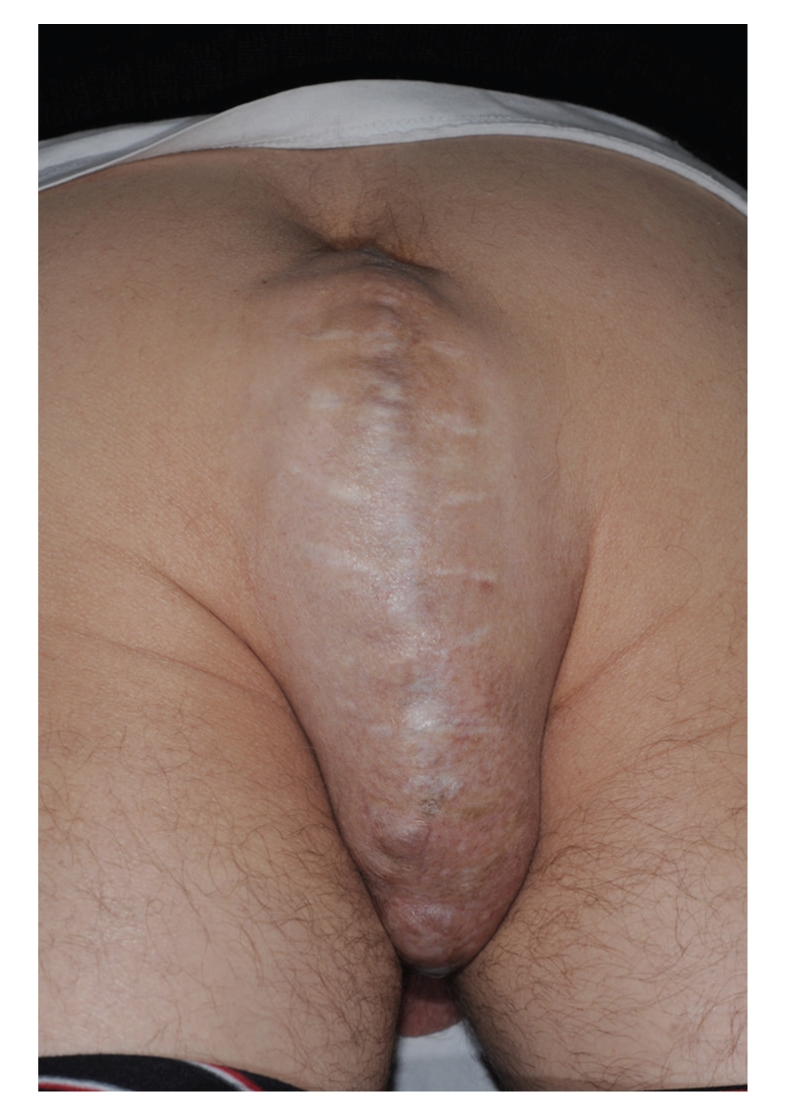
Preoperative picture showing the perineal hernia defect.

At six-month clinical followup, he complained of a reducible, painless incisional perineal hernia extending into the scrotum (Figure 1). He subsequently underwent elective laparoscopic mesh repair of this perineal hernia.

**Figure 2:**
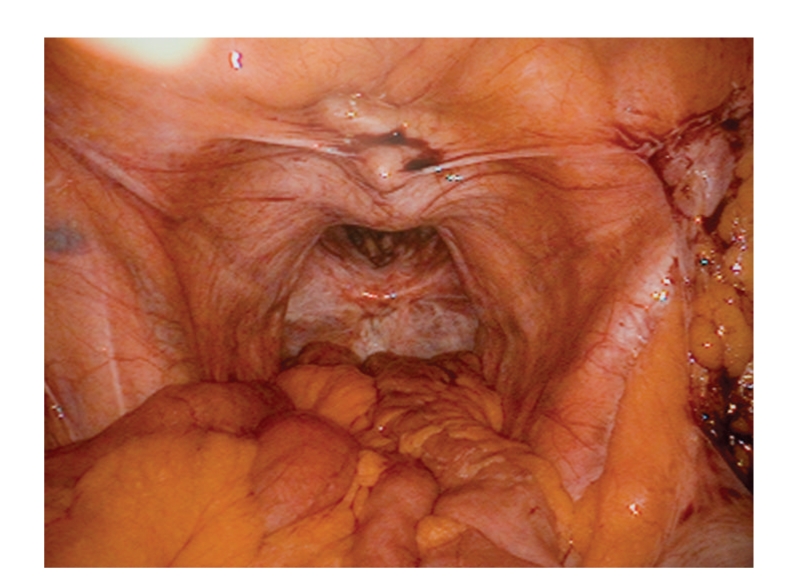
Intra-abdominal view of bowel loops mobilised from perineal wall defect.

The operation was performed in lithotomy position. A urethral catheter was placed to decompress the bladder. Pneumoperitoneum was established using the Hassan technique via a curvilinear infraumbilical incison. Three additional 5 mm ports were placed under direct vision using a 10 mm 0° laparoscope. One was positioned in the right lower quadrant, one in the right upper quadrant, and a third was placed in the left lower quadrant. At laparoscopy, there was no evidence of disease recurrence. Placement of the patient in a steep trendelenburg position facilitated division of adhesions with mobilisation of the small bowel loops out of the hernial defect (Figure 2). Both ureters were identified. The defect was repaired using a nonabsorbable composite mesh (Composix E/X Oval, 18 × 23 cms (Bard Nordic, Sweden) ellipse mesh, 7 × 9′′). The mesh was inserted through the 12 mm optical port and tacked anteriorly to the symphysis pubis and pelvic brim using a stapler device (StatTack, Autosuture, Tyco Healthcare UK Ltd). It was anchored to the sacrum using the endotacker. The remaining mesh was laparoscopically sutured to the surrounding pelvic brim and lateral abdominal wall, taking great care to avoid the ureters, iliac vessels and inferior epigastric vessels, using interrupted 3/0 vicryl sutures (Ethicon.Inc).

**Figure 3:**
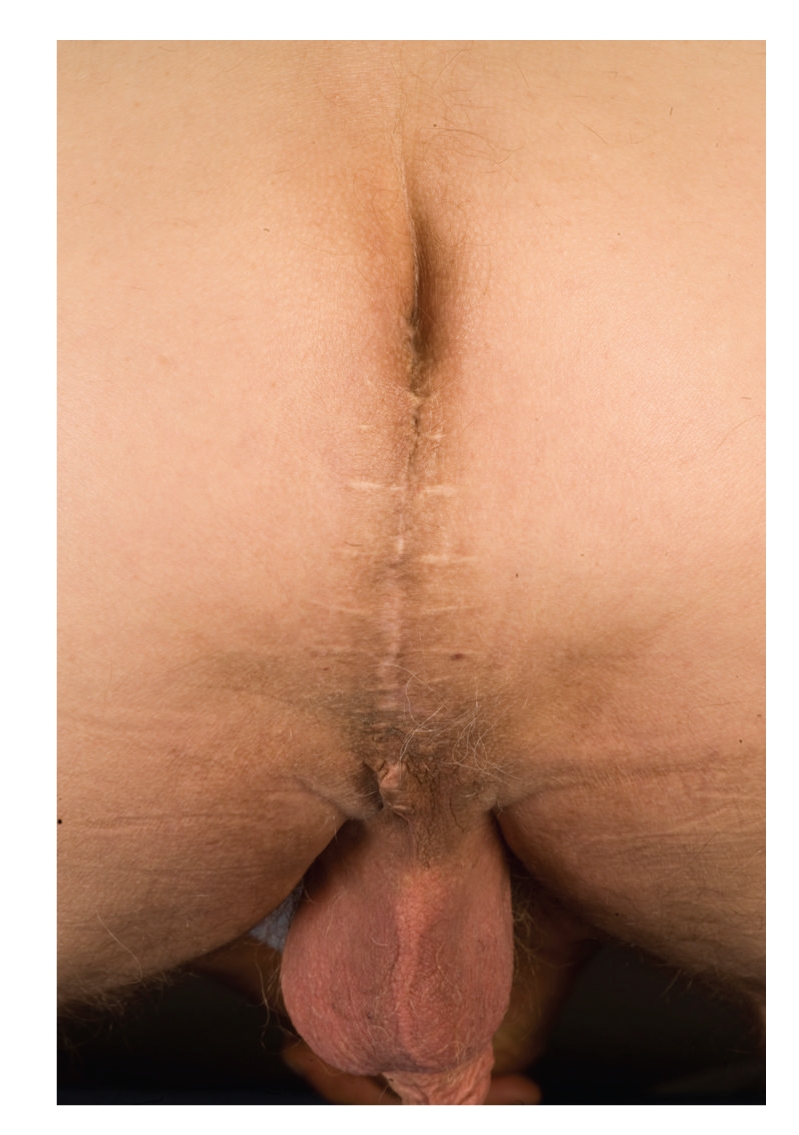
Five months postoperative perineal hernia repair with no evidence of recurrence.

The patient made an uneventful recovery and was discharged 2 days later. Following repair, the patient remained asymptomatic with no evidence of recurrence of the perineal hernia (Figure 3, five months post repair) at 18-month followup. 

## 3. Discussion

Perineal hernias may be classified as primary (congenital or acquired) or secondary (postoperative). They are infrequent complications of major pelvic surgery and when present are usually asymptomatic. Aboian et al. [1] recently showed in a retrospective study a prevalence rate of symptomatic postoperative perineal hernias of 0.34% with a more common prevalence associated with those who have had cancer operations. Smoking, female gender, and chemoradiotherapy are other important risk factors. The duration between surgery and hernia formation is usually between six months and five years [5]. The patient described in this case report had many of these risk factors including a rectal tumour, neoadjuvant chemoradiotherapy, and smoking.

Surgical repair of a postoperative perineal hernia is indicated if there is pain or discomfort, skin erosion over the herniated sac, or intestinal obstruction and involves mobilisation and reduction of the contents of the hernial sac with closure of the defect. Many techniques have been reported including transperineal, transabdominal and the combined abdominoperineal approach. Given the low prevalence of such hernias there is, however, no consensus as to which approach is best. Aboian et al. [1] in their review suggest that the abdominal approach has advantages that confer superiority over the transperineal option, with better exposure for dissecting out sac contents, hernial boundaries and pelvic contours. In addition, it also provides good access for mesh positioning and allows for exclusion of small bowel from the repair. Despite the increase use of laparoscopy as a surgical technique, to date there have been few reports of its application to repair of postoperative perineal hernias [9]. Laparoscopy has the advantage of quicker recovery time, faster recovery of bowel function, and decreased immunological stress while offering the same advantages as open abdominal surgery including good visualisation of intra-abdominal contents and the ability to survey for evidence of tumour recurrence intraoperatively [8]. In support of this, our results using laparoscopic repair demonstrate that it is an excellent alternative to other surgical repair techniques with good early postoperative outcomes. 

Various techniques to repair the defect in the pelvic floor have previously been used. These include synthetic mesh repair, omentoplasty, musculocutaneous rotation flaps, and free facia lata flaps [11, 12]. Nonabsorbable meshes, such as that used in this case report, are increasingly being used to provide a new pelvic floor in cases of large defects. The composite mesh which has a hydrophilic film reduces the risk of visceral adhesions while the nonresorbable polyester mesh provides long-term reinforcement of soft tissues [13]. Again, we have demonstrated good early results with the use of this mesh but further long-term followup is warranted.

In conclusion, symptomatic perineal hernias, which are rare complications of pelvic surgery, require surgical repair. Many approaches have previously been described. We have demonstrated that the use of laparoscopy with repair of the pelvic floor defect using nonabsorbable synthetic meshes offers an excellent alternative with many potential advantages over open transabdominal and transperineal repairs.
